# Molecular lipids in prediction of psychosis and the associated cardiometabolic co-morbidities

**DOI:** 10.1192/j.eurpsy.2021.66

**Published:** 2021-08-13

**Authors:** M. Oresic

**Affiliations:** School Of Medical Sciences, Örebro University, Örebro, Sweden

**Keywords:** lipidomics, psychosis, lipid metabolism, metabolic co-morbidities

## Abstract

Lipid metabolism has been an area of increased interest in psychosis research, not only due to its link to metabolic comorbidities, but also due to its putative role in the pathophysiology of psychosis. Lipid disturbances are observed already in the period preceding the onset of psychosis. For example, we performed mass spectrometry based lipidomics in a cohort of individuals at clinical high risk for psychosis (the EU-GEI study) and found that the individuals who transitioned to psychosis within a 2-year follow-up period displayed decreased levels of ether phospholipids. This finding may be of direct (patho)physiological relevance, as ether phospholipids (particularly plasmalogens, a major subgroup of ether phospholipids) are highly enriched in the brain, are supplied to the brain by the liver, have many structural and functional roles, and may act as endogenous antioxidants. Accumulating evidence also suggests that lipid disturbances play a crucial role in the development of metabolic comorbidities associated with psychotic disorders. Our lipidomic studies have shown that psychotic patients who rapidly gain weight during follow-up have elevated triglycerides (TGs) with low double bond count and carbon number at baseline. These TGs are known to be associated with non-alcoholic fatty liver disease (NAFLD) and with increased risk of type 2 diabetes. In conclusion, although the mechanisms linking dysregulation of lipid metabolism with the pathophysiology of psychosis are currently poorly understood, findings by us and others suggest that metabolic abnormalities are evident in people who are vulnerable to psychosis, and to the associated metabolic comorbidities.

**Disclosure:**

No significant relationships.

